# Bearing Fault Diagnosis Based on Energy Spectrum Statistics and Modified Mayfly Optimization Algorithm

**DOI:** 10.3390/s21062245

**Published:** 2021-03-23

**Authors:** Yuhu Liu, Yi Chai, Bowen Liu, Yiming Wang

**Affiliations:** 1 College of Automation, Chongqing University, Chongqing 400044, China; lyhu@cqu.edu.cn (Y.L.); liubowen@cqu.edu.cn (B.L.); cquawang1ming@cqu.edu.cn (Y.W.); 2 State Key Laboratory of Power Transmission Equipment and System Security and New Technology, Chongqing University, Chongqing 400044, China

**Keywords:** bearing fault, MMA, impulse signal, energy spectrum, fault diagnosis

## Abstract

This study proposes a novel resonance demodulation frequency band selection method named the initial center frequency-guided filter (ICFGF) to diagnose the bearing fault. The proposed technology has a better performance on resisting the interference from the random impulses. More explicitly, the ICFGF can be summarized as two steps. In the first step, a variance statistic index is applied to evaluate the energy spectrum distribution, which can adaptively determine the center frequency of the fault impulse and suppress the interference from random impulse effectively. In the second step, a modified mayfly optimization algorithm (MMA) is applied to search the optimal resonance demodulation frequency band based on the center frequency from the first step, which has faster convergence. Finally, the filtered signal is processed by the squared envelope spectrum technology. Results of the proposed method for signals from an outer fault bearing and a ball fault bearing indicate that the ICFGF works well to extract bearing fault feature. Furthermore, compared with some other methods, including fast kurtogram, ensemble empirical mode decomposition, and conditional variance-based selector technology, the ICFGF can extract the fault characteristic more accurately.

## 1. Introduction

Rolling bearings have been widely used in modern industry, especially in rotation machinery. The operation pattern of the bearing determines it is a worn part. More explicitly, bearings in the engine or high-speed railway wear easily because of the poor working environment and unstable load. Consequently, bearing fault is a hot research topic in rotation machinery fault diagnosis [1,2,3]. The vibration signal can reflect the health status of rotation machinery effectively [4] and has been widely applied in fault diagnosis [5,6,7]. However, the response of the early localized damage is too weak to be detected directly and is usually submerged by noises from the environment and other running components. Consequently, a trustworthy signal process technology must be carried out.

For decades, many methods have been proposed for bearing fault diagnosis. Envelope analysis is one of the most popular approaches and can be designed as two steps [8,9,10]. The first step is to determine the optimal resonance frequency band (ORFB). The next step is to process the signal filtered by the demodulation technology to obtain the squared envelope (SE) or the squared envelope spectrum (SES), which can display the fault feature much more clearly than the raw feature. The key point in envelope analysis is to find the ORFB with the help of a suitable index [11,12].

For bearing fault diagnosis, the most popular approach is fast kurtogram (FK), whose index is kurtosis [13]. However, FK has two drawbacks. One is the frame inflexibility of the filter-banks, and the other is the vulnerable index. Many researchers have focused on solving these two problems. For the former, wavelet packet transform (WPT) [14] and a sliding window filter (SLF) [15] were proposed to extract the fault feature. WPT and SLF can improve the flexibility for the frame of the filter-banks, but their indices still suffer from the incursion of random impulses. Therefore, more researchers have been devoted to improving the anti-noise performance of kurtosis. First, some indices, including Gini index [16], L2/L1-norm [17], and infogram-based index [18], have been proposed to replace the index in FK. The three above indices worked better in resisting Gaussian noise but were still weak when random impulses existed in the measured signal. To fill this gap, correlated kurtosis kurtogram [19] and periodicity-based kurtogram [20] were proposed to extract the bearing fault impulses. Another way to fill this gap is to calculate the kurtosis of the SES [21]. These three methods improved the performance on the resisting random impulses but neglected the frame’s flexibility of the filter-banks. Furthermore, some other methods, including the ratio of the cyclic content method [22], the harmonic-to-noise ratio method [23], and adaptive harmonic kurtosis [24], were proposed to diagnose the bearing fault. However, they have not gained much attention because of the necessity for the priori information. All of these above methods can be briefly summarized as follows. The methods with flexibility windows have vulnerable indices, and those with improved indices have rigidity windows. Consequently, some problems need to be solved for searching the ORFB.

To determine the ORFB accurately, a novel method based on a conditional variance-based (CVB) selector was proposed by Hebda-Sobkowicz et al. [25]. The CVB was applied to the time-frequency analysis result, which inherited the drawbacks of the short-time Fourier transform and was still weak to random impulses. To overcome these two drawbacks, a novel method named the initial center frequency-guided filter (ICFGF) is proposed in the present study. In the proposed method, we build a novel index based on the energy spectrum feature of impulse signal. Thus, the index can adaptively determine the center frequency of the impulse component and is powerful enough to resist the interferences from random impulses. The width for the ORFB is determined through a modified mayfly algorithm (MMA) in which the initial agents are designed based on the initial frequency. To verify the effectiveness of the proposed index, the simulation signal is repeated 1000 times with the same level of Gaussian noise. The result shows that the index works well to identify the center frequency of the impulse component. Furthermore, signals from an outer fault bearing and a ball fault bearing are applied to examine the reliability of the proposed method. Moreover, compared with FK, ensemble empirical mode decomposition (EEMD), and the CVB selector technology, the ICFGF works better to extract bearing fault feature. Consequently, the contributions of this paper can be summarized as follows: one is that a novel index is proposed to determine the resonant frequency for the bearing fault features; the other is that a new distribution mode for the initial agents is proposed in this paper.

The rest of this paper is arranged as follows. Section 2 introduces the proposed method, including the novel spectrum statistic index, the simulation result, and the MMA optimization algorithm. Section 3 shows the case study and comparison results. Section 4 arranges the concise conclusions.

## 2. The Proposed Method

This section briefly introduces some relative methods, including the conditional variance statistic index and the MMA optimization algorithm.

### 2.1. CV for Gaussian Data

The distribution for a set of Gaussian data can be described as

(1) $$X∼N(\mu ,\sigma^{2}),$$

where μ and $\sigma^{2}$ denote the mean and variance, respectively. From Hebda-Sobkowicz et al. [25] and Jaworski and Pitera [26], the conditional variance can be defined as

(2) $$\sigma_{A}^{2}=var\left(X|X\in A\right).$$

**Figure 1:**
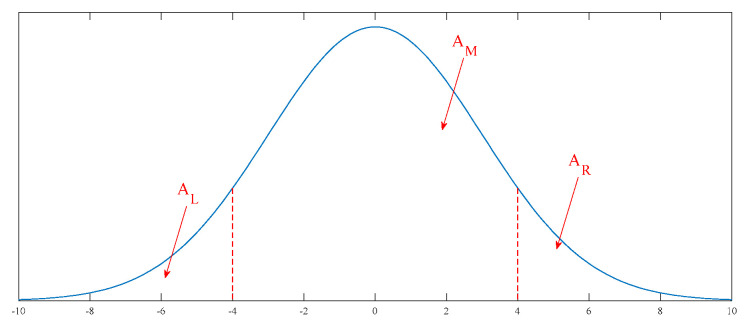
Sample for 20/60/20: area left (A_L_), area middle (A_M_), and area right (A_R_) corresponding to 20%, 60%, and 20%, respectively.

Based on Jaworski and Pitera [26], Gaussian distribution can be divided into three parts, and each of them has the same variance. The ratio for each part is 20/60/20, which is displayed in Figure 1. 

**Figure 2:**
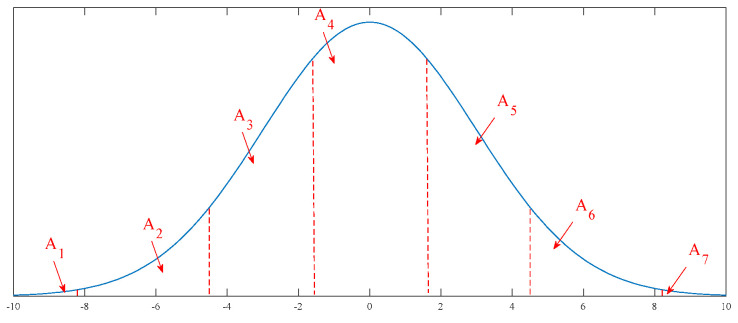
Sample for seven parts: A_1_, A_2_, A_3_, A_4_, A_5_, A_6_, and A_7_ corresponding to 0.4%, 5.8%, 24.6%, 38.4%, 24.6%, 5.8%, and 0.4%, respectively.

Furthermore, Hebda-Sobkowicz et al. [25] and Jelito and Pitera [27] proposed to divide the distribution into seven parts, which is shown in Figure 2. The ratio for each is designed as 0.4/5.8/24.6/38.4/24.6/5.8/0.4. Similar to the 20/60/20 principle, the variance for each part is also equivalent.

The power spectral density of Gaussian noise is a constant, which means the energy of Gaussian noise for the whole frequency domain should also be a constant. In practice, Gaussian noise will be filtered by the collection equipment, which means its energy spectrum will be presented as a Gaussian distribution. Based on the inheritance of data, a sub-part from the energy spectrum is also a Gaussian distribution. However, the energy for the impulse component will be concentrated on a series of resonance spectrum lines, which means its energy spectrum is not a Gaussian distribution. Consequently, we can distinguish it from the energy spectrum.

To identify the impulse component, a novel index is proposed in this study. Firstly, the signal is processed by FFT technology. Next, the square operation is applied to transform the FFT spectrum to the energy spectrum. Then, to suppress the effect from a harmonic component, areas A_1_ and A_7_ shown in Figure 2 are abandoned. Finally, by using the result of 20/60/20 for the remaining data, the index can be calculated as

(3) $$CV=\left(\sigma_{A_{R}}^{2}-\sigma_{A_{M}}^{2}\right)\times \sigma_{A_{R}}^{2}\times N,$$

where $A_{R}$ and $A_{M}$ denote the areas shown in Figure 1, and N is the length of the data analyzed. From Equation (3), the value of CV should be small if the data to be analyzed are Gaussian. CV should be large for the impulse component.

In this study, CV is combined with sliding technology. Consequently, the length of the window and its step size must be determined first. In this study, we suggest that the length of the window should meet the following conditions:

(1) The length can divide the whole energy spectrum into 50–100 sub-parts, and the center frequency for each sub-part is mark as $f_{c}$.
(2) The length of the window should be wide enough to contain at least three fault feature spectrum lines. Considering the unknown fault type, the length can be set as two times larger than the inner fault feature frequency.
(3) The overlap ratio between two windows should be more than 50%. In this study, the overlap ratio is set as 80%.

To verify the effectiveness of CV and the sliding window strategy, the simulation signal is designed as

**Figure 3:**
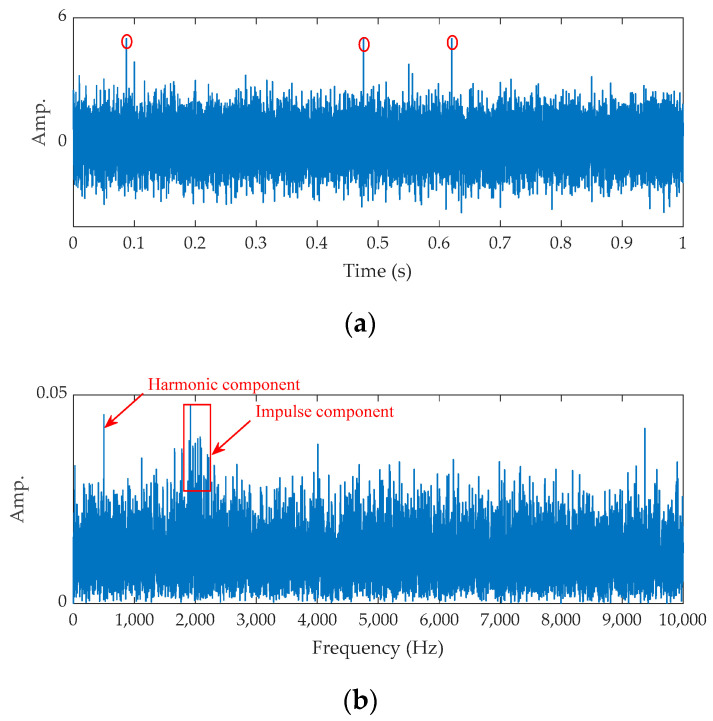

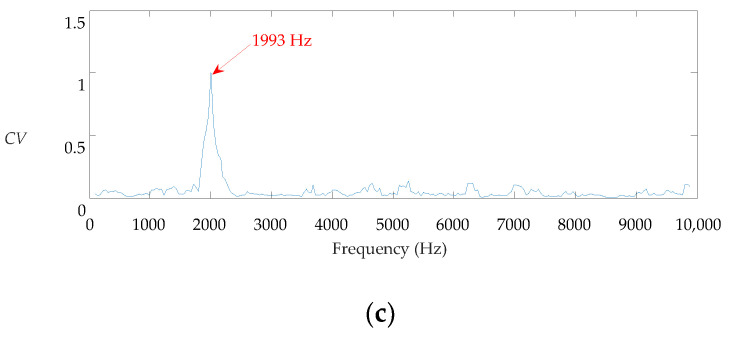
The results for the simulation signal: (**a**) time waveform, (**b**) FFT spectrum, and (**c**) $CV\;\mathrm{vs}.\;f_{c}$.

(4) y(t)=I(t)+0.04×sin(2π×500×t)+n(t)

(5) $$I\left(t\right)=2\times e^{0.1\times 2\pi \times 2000}{}^{t}\sin\left(2\pi \times 2000t\times \sqrt{1-0.1^{2}}\right),$$

where the first item on the right of Equation (4) denotes the impulse component with a 20 Hz period. The second denotes the harmonic component, and the last item denotes Gaussian noise with 0.4 intensity. Three impulse interferences are added into the simulation signal and marked by red circles. The corresponding time waveform and FFT spectrum are shown in Figure 3. Based on Figure 3b, the impulse component is interfered by the noise seriously. The proposed energy spectrum statistic index is applied to process these data, and the result is shown in Figure 3c. From it, the frequency of the max CV is 1993 Hz, which is close to the design frequency. An important thing to keep in mind is that CV is normalized in the whole study.

**Figure 4:**
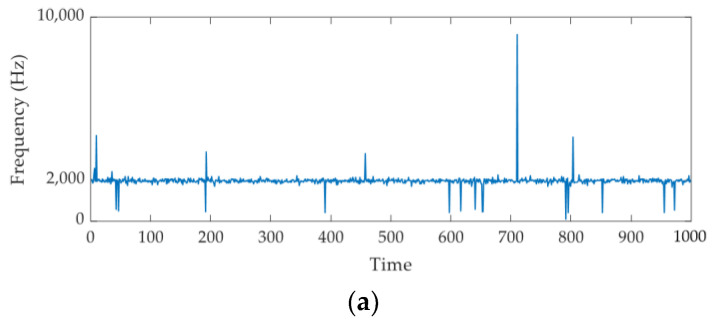

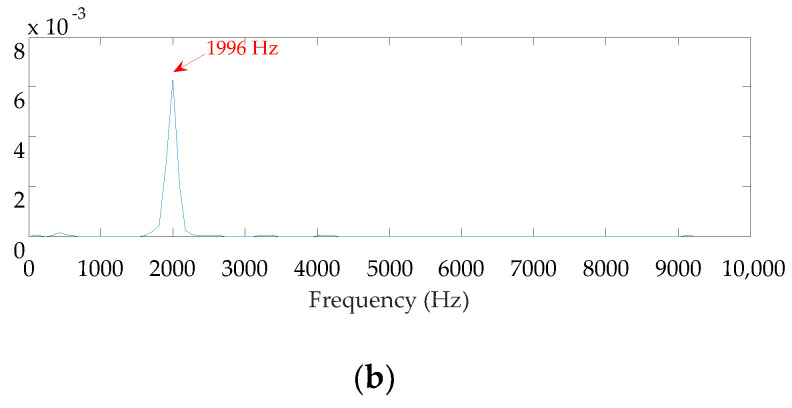
The results of CV index for the 1000 simulation signals: (**a**) $f_{c}$ and (**b**) the probability density estimate for $f_{c}$.

To verify the effectiveness of the index further, Equation (4) is repeated 1000 times to obtain 1000 simulation signals. The 1000 signals are analyzed by the CV index, and the results are shown in Figure 4. As shown in Figure 4a, most of the results are close to the designed resonant frequency, and the density curve of these results displayed in Figure 4b verifies this result again. More specifically, only 23 points are located outside the range [1700–2300]. Consequently, the new index can identify the center frequency of the impulse component effectively.

### 2.2. The MMA Optimization Algorithm

The center frequency of the ORFB can be obtained by the index shown in the previous section. In this current section, we determine the bandwidth for the ORFB through an MMA optimization algorithm.

The mayfly algorithm (MA) was first proposed by Zervoudakis and Tsafarakis [28]. Its main steps can be summarized as follows.

(1) Initialization: The populations of males and females are first initiated as $x=\left[x_{1},\cdots ,x_{d}\right]$ and $y=\left[y_{1},\cdots ,y_{d}\right]$, respectively. The corresponding velocity is $v=\left[v_{1},\cdots ,v_{d}\right]$.
(2) Males’ movement: The global best position for the current iteration is determined by the object function f(x), which is marked as f(gbest). The position for the next iteration is updated based on f(gbest) and the Cartesian distance between the personal element and the global best agent gbest, which can be described as
  (6) $$x_{i}^{t+1}=x_{i}^{t}+v_{i}^{t+1}$$
  (7) $$v_{ij}^{t+1}=v_{ij}^{t}+a_{1}e^{-\beta r_{g}^{2}}\left(gbest_{j}-x_{ij}^{t}\right)+a_{2}e^{-\beta r_{p}^{2}}\left(pbest_{ij}-x_{ij}^{t}\right),$$
  where $x_{ij}^{t}$ corresponds to the agent i in dimension j at the current iteration t and $v_{ij}^{t}$ denotes its velocity. $a_{1}$ and $a_{2}$ denote the global and personal learning coefficient, respectively. $r_{g}$ and $r_{p}$ denote the Cartesian distance for global and personal, respectively. The velocity of the best agent in the current iteration is updated as $v^{t+1}=v^{t}+d\times r$, where d denotes the nuptial dance and r is a random variable located in [−1,1].
(3) Females’ movement: Compared with the movement of males, females will fly to males for breeding. Thus, their velocities are updated based on the Cartesian distance between themselves and the males, which can be described as
  (8) $$v_{ij}^{t+1}=\left\{ \begin{array}{l} v_{ij}^{t}+a_{3}e^{-\beta r_{mf}^{2}}\left(x_{ij}^{t}-y_{ij}^{t}\right),\;\mathrm{if}\;f\left(y_{i}\right)>f\left(x_{i}\right)\\ v_{ij}^{t}+fl\times r,\;\mathrm{if}\;f\left(y_{i}\right)\leq f\left(x_{i}\right) \end{array}\right.,$$
  where y corresponds to the female agents and $a_{3}$ denotes the learning coefficient. β is the distance sight coefficient, which is a constant. $r_{mf}$ denotes the Cartesian distance between the male and female agents. The best female is arranged to match with the best male, and the second-best female is matched to the second-best male. Consequently, the positions of males are very important in MA.
(4) Mating: Each couple produces two offspring in MA. One of them is added to the male population randomly, and the other is added to the female population.
(5) Updating: The worst solution is replaced by the best solution, and the processes above are repeated until the stop criteria are met.

Based on the description above, understanding that the position of the male population is the key point in MA is easy. Their positions are initialized randomly in the original MA. However, in this study, we can design a specific initiation process based on the result of the energy spectrum statistic index, which can adjust the distribution of the males’ positions corresponding to center frequencies reasonably. In this study, the number of agents is 10 for both the male population and female population. The range for the center frequency is $\left[0.85\times f_{c},1.15\times f_{c}\right]$, where $f_{c}$ denotes the center frequency obtained by the CV index, and the range for the bandwidth is [BSF,4×BPFI], in which BSF denotes the feature frequency of ball fault and BPFI denotes the feature frequency of inner fault. Motivated by the 20/60/20 principle in the previous section, males are arranged as follows: six (60%) males are in $\left[0.95\times f_{c},1.05\times f_{c}\right]$, two (20%) are in $\left[0.85\times f_{c},0.95\times f_{c}\right]$, and the remainder (20%) are arranged in $\left[1.05\times f_{c},1.15\times f_{c}\right]$.

### 2.3. The Proposed Method

Before introducing the proposed method, the optimization function must be determined. In this study, weight kurtosis is set as the objection function and can be calculated as

(9) CK=C×K,

where C denotes the correlation coefficient between the filtered signal and the raw signal, and K denotes the kurtosis of the filtered signal. Given that the goal of MA is to determine the global minimum, the objective function in MMA should be set as −CK.

The main steps of the proposed method can be summarized as follows:

Step 1: The measured signal is first processed by FFT technology, and square operation is also applied to transform the FFT spectrum into an energy spectrum.

Step 2: The energy spectrum statistic index CV is applied to evaluate the center frequency for the ORFB.

Step 3: The width of the ORFB is determined by mining −CK with the MMA optimization algorithm.

Step 4: SES technology is applied to analyze the signal filtered with the optimal ORFB.

**Figure 5:**
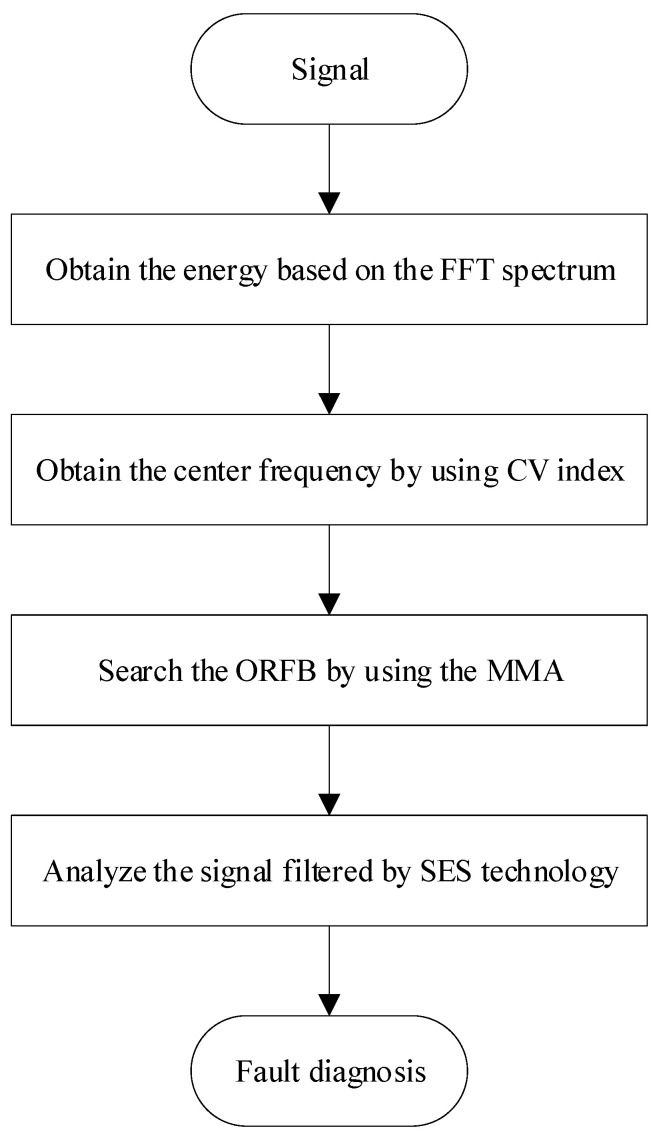
The initial center frequency-guided filter (ICFGF) flowchart.

To understand the proposed method comfortably, the flowchart of ICFGF is shown in Figure 5.

## 3. Case Study

This section applies signals from an outer fault bearing and a ball fault bearing to verify the effectiveness of the proposed method. The highlight of the proposed method is shown by comparing with some existing methods, including FK, EEMD, and the CVB index technology.

### 3.1. Inner Fault Diagnosis

**Figure 6:**
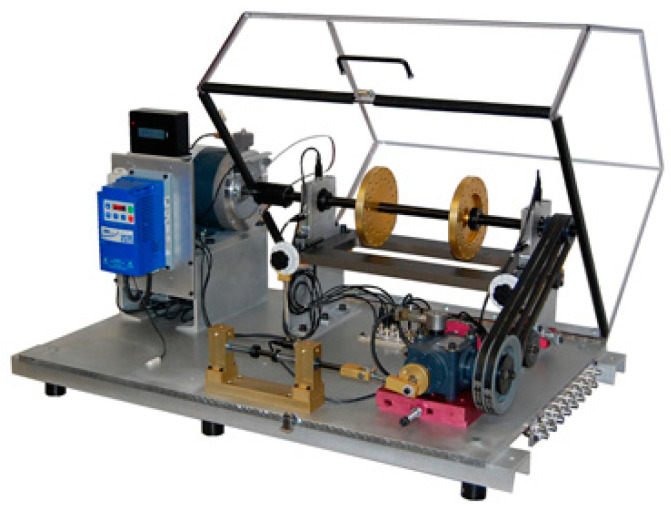
The test rig of the outer fault bearing.

The data come from Curtin University and have been applied in Qin et al. [24]. The signal is produced by a machinery fault simulator, which is displayed in Figure 6.

The signal is collected by an accelerometer with the sample frequency of 51.2 kHz. The test bearing is MB ER-16K, and there is a local defect which exists in its outer race. The test shaft speed is 1740 rpm, and the corresponding ball pass frequency outer race (BPFO) is 103.6 Hz. The length of the signal applied in this part is 1 s.

**Figure 7:**
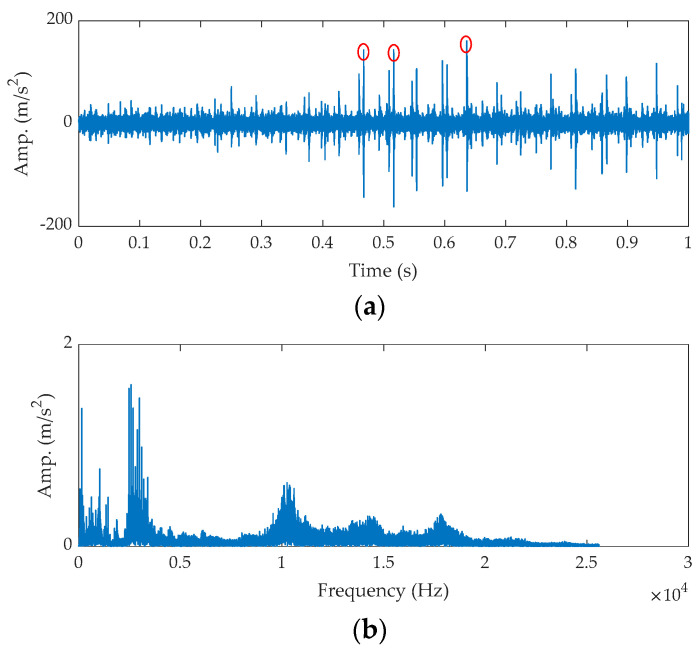
The raw signal for the outer fault bearing: (**a**) time waveform and (**b**) FFT spectrum.

The time waveform of the raw signal and its fast Fourier transform (FFT) spectrum are shown in Figure 7. As shown in Figure 7a, some random impulses (marked by red circles) are found. The FFT spectrum shown in Figure 7b tells us the fault feature is submerged by noise. Next, the proposed method is applied for the analysis.

**Figure 8:**
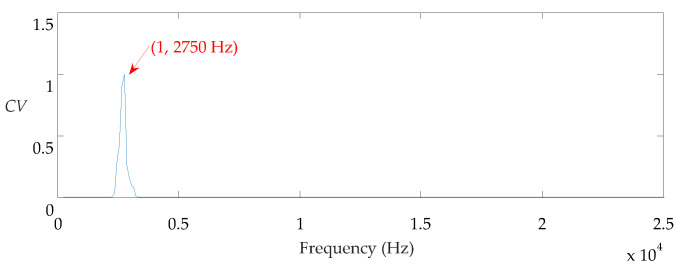
CV vs. $f_{c}$ for the outer fault signal.

The center frequency is first evaluated by the CV index, and the result is shown in Figure 8. The center frequency is 2750 Hz. Then, the MMA is applied to find the ORFB. In this case, the range for searching center frequency is set as [2337, 3162] Hz, and the range for searching bandwidth is set as [124, 628] Hz. From the MMA, the center frequency of ORFB is 2806 Hz and the corresponding bandwidth is 628 Hz.

**Figure 9:**
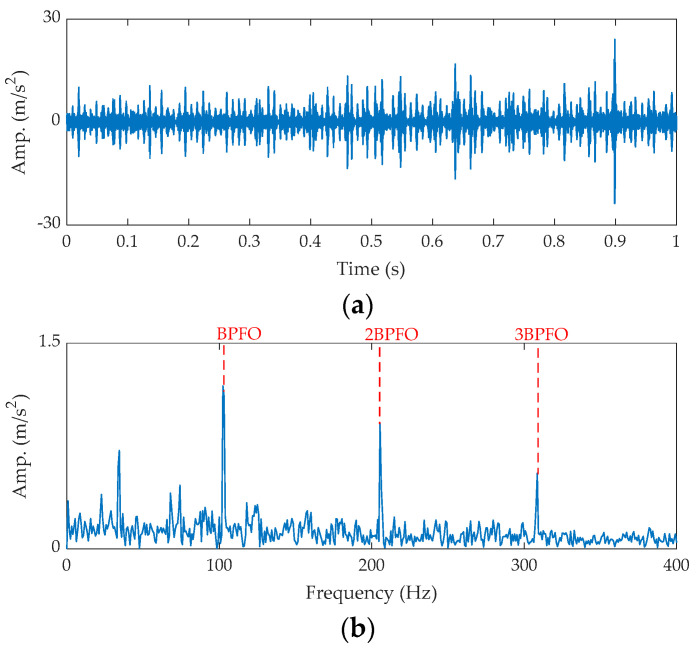
The results by ICFGF for the outer fault signal: (**a**) filtered signal and (**b**) SES.

The results are shown in Figure 9. From Figure 9a, the periodic impulses can be observed easily. Its SES depicted in Figure 9b shows the fault features (BPFO, 2BPFO, and 3BPFO) clearly.

**Figure 10:**
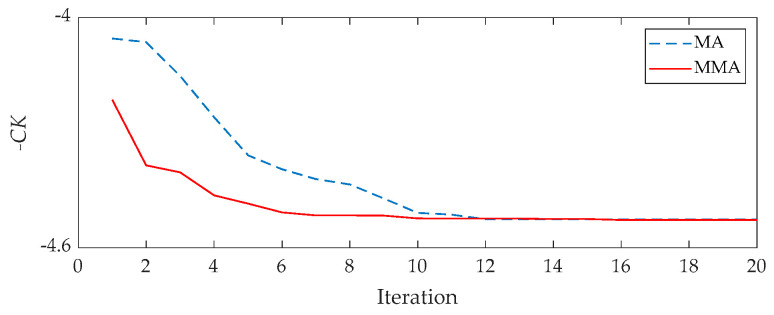
The results of every iteration for the outer fault signal: red line for the MMA and blue line for the raw MA.

Both MMA and MA are re-run three times to show the superiority of MMA. Their average results are shown in Figure 10. From it, we can say the MMA has a faster convergence. 

**Figure 11:**
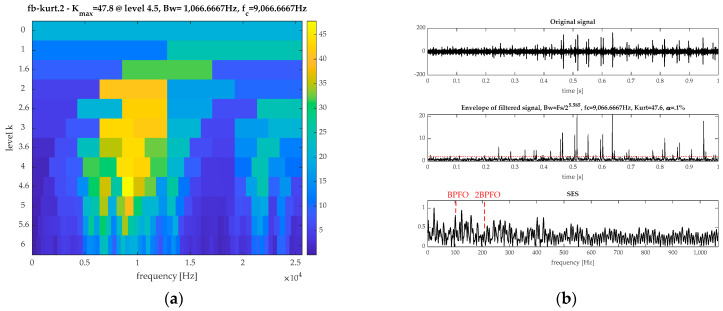
The results by FK for the outer fault signal: (**a**) spectrum kurtosis and (**b**) the filtered signal and its SES.

To highlight the superiority of the ICFGF, the outer fault signal is also processed by FK, EEMD, and CVB technology. The results by FK are displayed in Figure 11. From Figure 11a, the ORFB is [8533, 9599] Hz. The filtered signal and the corresponding SES are shown in Figure 11b, which illustrates that the SES is complex and the fault feature is interfered by the noise.

**Figure 12:**
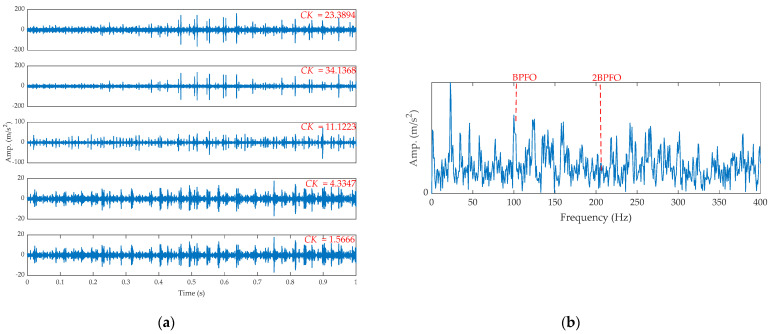
The results by EEMD for the outer fault signal: (**a**) intrinsic mode function (IMF) and (**b**) the SES of the second IMF.

Figure 12 displays the results of EEMD. From Figure 12a, the second sub-signal has the max CK, and its SES is shown in Figure 12b. From Figure 12b, many interference components are found, and the feature is not as clear as the results shown in Figure 9b.

**Figure 13:**
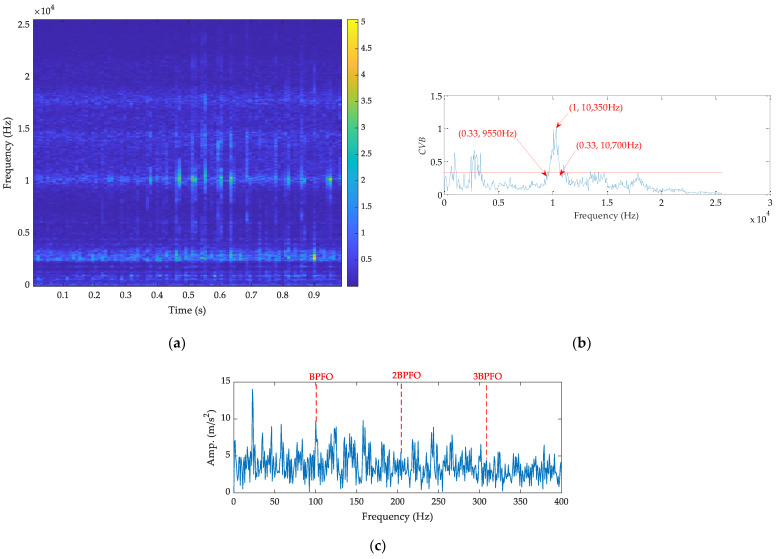
The results by CVB technology for the outer fault signal: (**a**) time–frequency spectrum, (**b**) *CVB* vs. frequency, and (**c**) SES.

Finally, the outer fault signal is processed by the CVB technology, and the results are shown in Figure 13. From Hebda-Sobkowicz et al. [25], the ORFB is determined by the threshold value technology, which is a third smaller than the max value and is marked by the red line. Based on this, the ORFB is [9550, 10,700] Hz. The corresponding SES is shown in Figure 13c, illustrating that the fault feature is still interfered by the noise, and a large gap exists between it and Figure 9b.

### 3.2. Ball Fault Diagnosis

**Figure 14:**
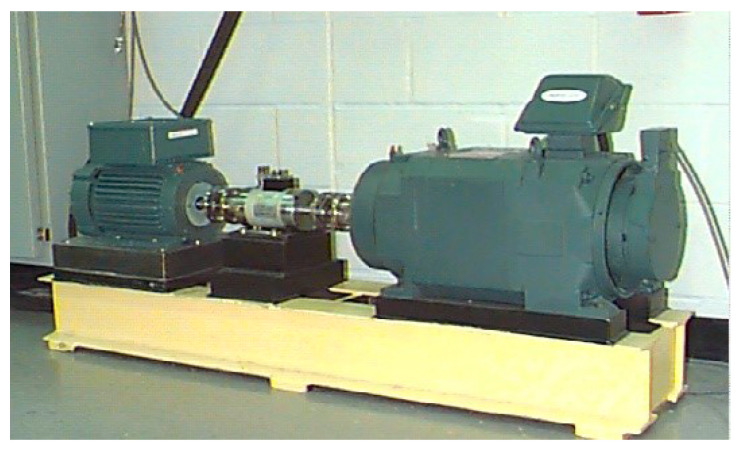
The test rig for the ball fault bearing.

The data come from the Case Western Reserve University Bearing Data Center Website [29] and had been applied in [1]. The test rig is displayed in Figure 14. The test ball fault bearing is 6205-2RS JEM SKF, which is installed at the drive end. The vibration data are collected by an accelerometer which is near the test bearing. The sample frequency is 48 kHz and the length of the signal used in this study is 1 s. The ball spin frequency (BSF) is 70.3 Hz because the shaft rotation speed is 1797 rpm. Importantly, the feature frequency of the ball fault should be 140.6 Hz because the local defect of the ball will touch with the inner race and outer race alternately.

**Figure 15:**
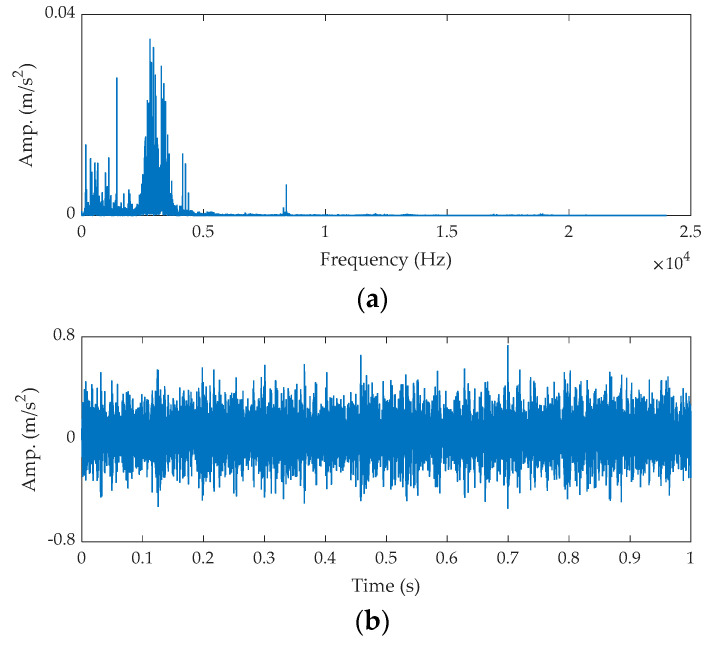
The raw signal of the ball fault bearing: (**a**) time waveform and (**b**) FFT spectrum.

The time waveform of the raw signal and its FFT spectrum are shown in Figure 15. 

**Figure 16:**
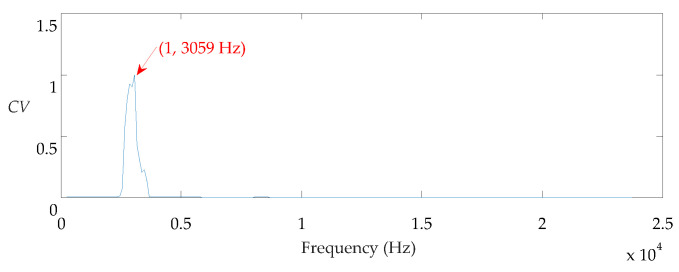
CV vs. $f_{c}$ for the ball fault signal.

The impulse component cannot be directly observed in both. The proposed method is applied to process this signal. First, the center frequency of the ORFB is 3059 Hz based on the *CV* index, which is shown in Figure 16.

**Figure 17:**
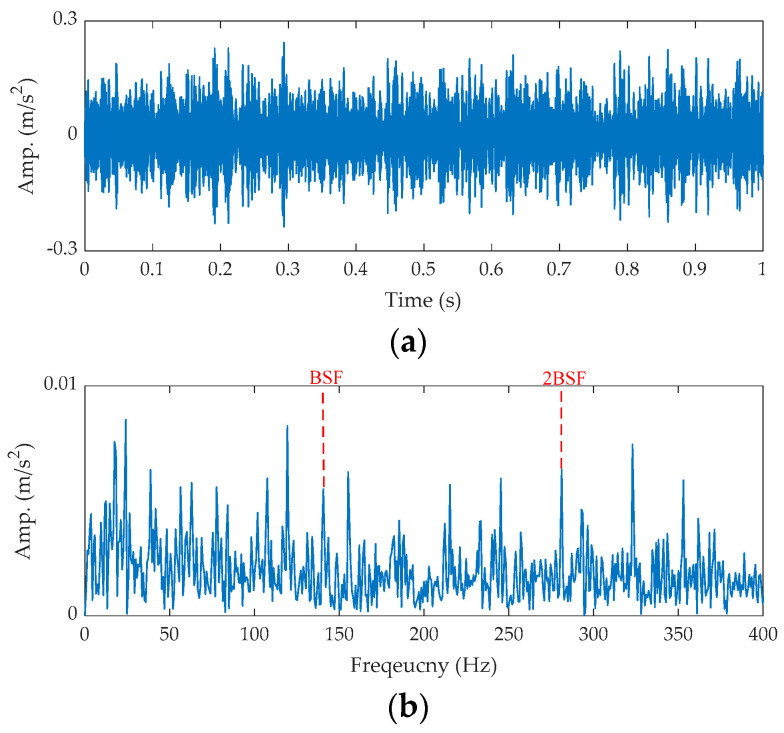
The results by the ICFGF for the ball fault signal: (**a**) filtered signal and (**b**) SES.

In this case, the range for searching center frequency is set as [2600, 3517] Hz and the range for bandwidth is [140, 649] Hz. The results from the MMA are 3087 Hz and 648 Hz. The former corresponds to the center frequency, and the latter corresponds to the bandwidth. The results are displayed in Figure 17. From Figure 17b, the fault feature and its harmonic BSFs are easily observed.

**Figure 18:**
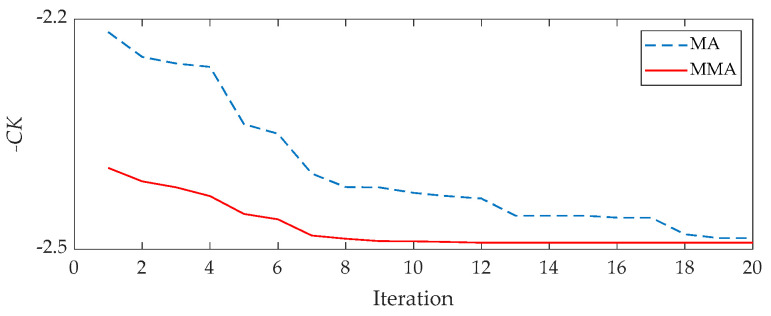
The results of each iteration for the ball fault signal: red line for the MMA and blue line for the raw MA.

Figure 18 displays the average results for the MMA and the raw MA. From it, MMA still has a faster convergence while processing the ball fault signal. Consequently, the proposed method works well to extract the bearing fault feature.

**Figure 19:**
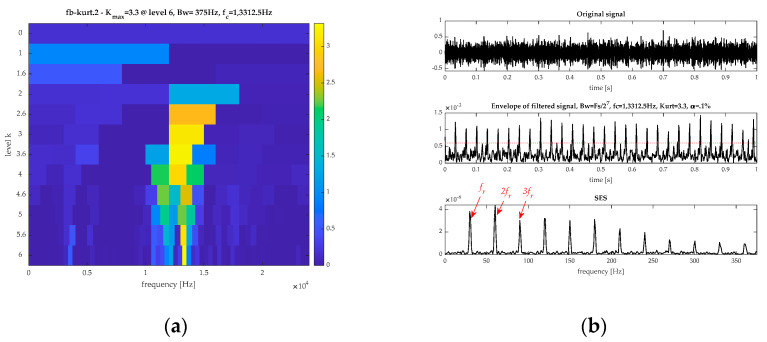
The results by FK for the ball fault signal: (**a**) spectrum kurtosis and (**b**) the filtered signal and its SES.

Similar to the last part, the signal is also analyzed by FK, EEMD, and CVB technology. Figure 19 displays the results from FK. Based on Figure 19a, the ORFB is [13,124, 13,499] Hz. The corresponding filtered signal and its SES are shown in Figure 19b. The SES is very clear. However, only the rotating frequency ($f_{r}$) and its harmonics can be found in it. Consequently, FK fails to extract the fault feature.

**Figure 20:**
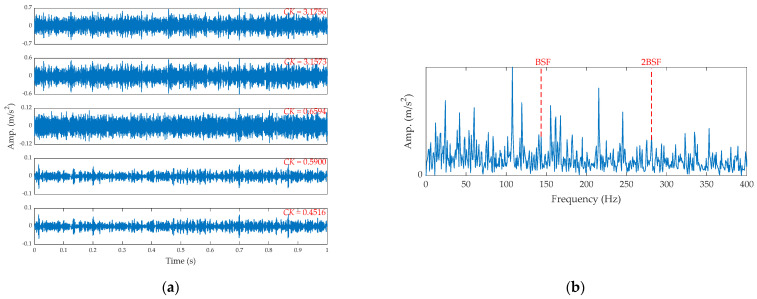
The results by EEMD for the ball fault signal: (**a**) IMFs and (**b**) the SES of the first IMF.

The EEMD is applied to analyze the signal, and the results are shown in Figure 20. From Figure 20a, the first sub-signal has the max CK. Its SES is shown in Figure 20b. It is very difficult to observe the fault feature due to the existence of noise.

**Figure 21:**
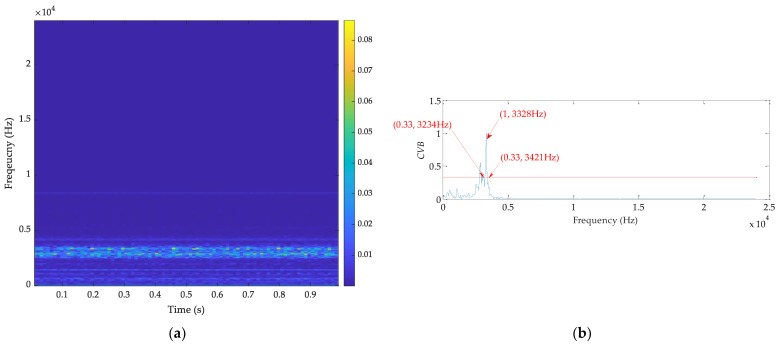

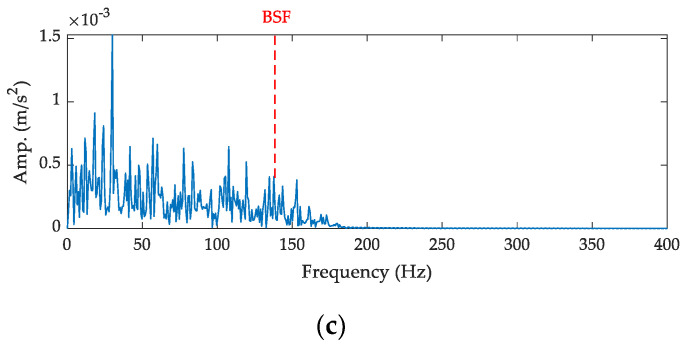
The results by CVB technology for the outer fault signal: (**a**) time–frequency spectrum, (**b**) *CVB* vs. frequency, and (**c**) SES for the filtered signal.

Finally, CVB technology is used to extract the fault feature, and the results are shown in Figure 21. From Figure 21b, the ORFB should be [3234, 3421] Hz. The SES for the filtered signal is shown in Figure 21c, which illustrates the difficulty in observing the fault feature. Therefore, the three methods cannot catch up with the performance for extracting the bearing fault feature of the proposed method.

## 4. Conclusions

To extract the bearing fault feature, this study proposes a new method to determine the ORFB, which includes a novel energy spectrum statistic index and an MMA optimization algorithm. The novel spectrum index is built based on the differences between the energy spectrum of Gaussian noise and the energy spectrum of the periodic impulses. The energy spectrum statistic technology is applied to evaluate the distribution of the energy spectrum, which can suppress the interference from the harmonic component effectively. Thus, the center frequency of the ORFB can roughly be obtained by using the energy spectrum statistic index. Then, the MMA optimization algorithm, which has fast convergence, is proposed to determine the ORFB. Finally, the fault feature is obtained by SES technology. The effectiveness of the proposed method is verified by the signals from an outer fault bearing and a ball fault bearing. Furthermore, comparing it with FK, EEMD, and CVB technology, our method better extracts the bearing fault feature from a heavy noise signal. Consequently, this study offers a new method to extract the bearing fault feature, especially for a processing signal which includes non-cyclic impulse interferences (i.e., crushing machinery).

## Data Availability

Not applicable.
